# Rapid amelioration of respiratory parameters in severely obese patients after percutaneous dilatational tracheotomy

**DOI:** 10.1186/cc14298

**Published:** 2015-03-16

**Authors:** S Kaese, M Zander, J Waltenberger, P Lebiedz

**Affiliations:** 1 University Hospital of Muenster, Münster, Germany

## Introduction

Incidence of obesity in developed countries is rising. Currently, Europe has a prevalence of 9 to 30% with significant impact on public health systems. Obese patients in the ICU require special management and treatment. Altered anatomy in obese patients complicates procedures such as mechanical ventilation. Obesity affects cardiopulmonary physiology and requires elevated ventilation pressures. In our retrospective study, we determined the effect of early percutaneous dilatational tracheotomy (PDT) and cessation of sedation on respiratory parameters in severely obese patients.

## Methods

From June 2010 to July 2014, we included all patients with a body weight of >130 kg, respiratory failure and PDT who were admitted to the ICU of the University Hospital of Muenster. We compared respirator parameters and blood gas analysis before and after PDT. Parameters were recorded on days -1, 0, 1, 3, 5, and 10, with day 0 describing the day of PDT.

## Results

Twenty-one patients were included in the study. Mean age was 56 ± 10.3 years and 14 (66.7%) of the patients were male. Body weight was 164.5 ± 39.4 kg, body height accounted for 176.8 ± 8.7 cm (*n *= 20) and body mass index was 49.7 ± 16.9 kg/m^2^. Patients stayed in the ICU for 18.4 ± 13.8 days. Mean time of mechanical ventilation by endotracheal tube was 2.4 ± 1.5 days (*n *= 20) and via tracheostomy 9.8 ± 7.0 days. After PDT, peak inspiratory pressure (*P *< 0.0001), positive end-expiratory pressure (*P *< 0.0001) and insufflated oxygen concentration (*P *< 0.0001) could significantly and rapidly be reduced. Respiratory minute volume increased significantly (*P *= 0.004). PDT was not associated with relevant complications.

## Conclusion

Early PDT rapidly improves respiratory distress in severely obese patients due enabling of spontaneous breathing and reduction of dead space ventilation.

